# Agri-Food Waste from Apple, Pear, and Sugar Beet as a Source of Protective Bioactive Molecules for Endothelial Dysfunction and Its Major Complications

**DOI:** 10.3390/antiox11091786

**Published:** 2022-09-10

**Authors:** Cristiana Caliceti, Marco Malaguti, Luisa Marracino, Maria Cristina Barbalace, Paola Rizzo, Silvana Hrelia

**Affiliations:** 1 Department of Biomedical and Neuromotor Sciences, Alma Mater Studiorum, University of Bologna, 40126 Bologna, Italy; 2 Interdepartmental Centre for Renewable Sources, Environment, Sea and Energy (CIRI FRAME), Alma Mater Studiorum, University of Bologna, 40126 Bologna, Italy; 3 Biostructures and Biosystems National Institute (INBB), 00136 Rome, Italy; 4 Department for Life Quality Studies, Alma Mater Studiorum, University of Bologna, 47921 Rimini, Italy; 5 Department of Translational Medicine and Laboratory for Technologies of Advanced Therapies (LTTA), University of Ferrara, 44121 Ferrara, Italy; 6 Maria Cecilia Hospital, GVM Care & Research, 48033 Cotignola, Italy

**Keywords:** endothelial dysfunction, agri-food waste, apple, pear, sugar beet, cardiovascular diseases

## Abstract

Endothelial damage is recognized as the initial step that precedes several cardiovascular diseases (CVD), such as atherosclerosis, hypertension, and coronary artery disease. It has been demonstrated that the best treatment for CVD is prevention, and, in the frame of a healthy lifestyle, the consumption of vegetables, rich in bioactive molecules, appears effective at reducing the risk of CVD. In this context, the large amount of agri-food industry waste, considered a global problem due to its environmental and economic impact, represents an unexplored source of bioactive compounds. This review provides a summary regarding the possible exploitation of waste or by-products derived by the processing of three traditional Italian crops—apple, pear, and sugar beet—as a source of bioactive molecules to protect endothelial function. Particular attention has been given to the bioactive chemical profile of these pomaces and their efficacy in various pathological conditions related to endothelial dysfunction. The waste matrices of apple, pear, and sugar beet crops can represent promising starting material for producing “upcycled” products with functional applications, such as the prevention of endothelial dysfunction linked to cardiovascular diseases.

## 1. Introduction

The adult population has an increasing incidence of hypercholesterolemia, diabetes, hypertension, and obesity. Among the major risk factors identified by the World Health Organization, unbalanced eating habits, as well as physical inactivity and high alcohol and tobacco consumption, are the most relevant [1].

Endothelial damage is a crucial aspect of atherosclerosis and precedes the overt manifestation of the disease. In addition, endothelial dysfunction is associated with hypertension, diabetes, ischemia–reperfusion injury, and neurodegenerative diseases [2,3]. It has been demonstrated that the best treatment for cardiovascular disease (CVD) is prevention, and diets containing greater quantities of vegetables appear effective at reducing the risk of CVD [4].

There is a great deal of epidemiological evidence indicating that a diet rich in fruit and vegetables is associated with a reduced risk of many chronic diseases, especially CVD [5,6]. The scientific community is highlighting the important action of natural molecules that can improve biological function and personal well-being [7,8].

Historically, waste from the food chain has been mainly exploited in the production of animal feed [9]. Only in recent decades, however, have these by-products started to be considered a resource to be valorized also in other industrial contexts [10,11]. More recently, studies and projects have begun to analyze and exploit the enormous number of bioactive compounds, still present in by-products, for the development of products with health-promoting properties for human use (foods, cosmetics, supplements, etc.) [12,13]. In this context, by-products from many plants have been studied, demonstrating that they provide health-promoting properties, such as antioxidant and chemopreventive ones [14,15,16].

In northern Italy, traditional apple, pear, and sugar beet crops are still considered among the most high-yielding crops in terms of harvesting, with a large production of by-products during their processing [17].

However, differently from by-products from grapevines, olive, orange, tomato, and chestnut [18,19,20,21,22,23], they are not very well studied for their bioactive content.

The study of suitable and eco-sustainable conditions to prevent the degradation of bioactive compounds and increase their yield of extraction is a crucial point to focus on.

In this review, we point out the possible role of the bioactive molecules present in pomace derived from the agri-food chains of apple, pear, and sugar-beet as compounds potentially beneficial for endothelial dysfunction, a key stage of several cardiovascular diseases (CVD).

## 2. Literature Search Strategy

The literature search was initially conducted on scientific literature databases (PubMed, Scopus, Google Scholar, and Web of Science) focusing on the following MeSH terms: endothelial dysfunction, cardiovascular diseases, apple, pear, sugar beet, waste, by-products, pomace. Peer-reviewed papers had to be written in English, available in full text, and published between 2002 and 2022. Papers published before 2002 were included if they were relevant in the field. We searched for research articles, reviews or editorials. Numerous articles were obtained, some of which did not precisely meet the field of interest; therefore, screening was undertaken by five independent authors. Disagreement was resolved by discussion and, if required, by a sixth independent author.

## 3. Endothelial Dysfunction and Its Major Complications

The endothelium is a single layer of endothelial cells that is not just lining the blood vessels but is actively involved in maintaining vascular health. It is considered the largest autocrine–paracrine organ of the body, and it plays both a passive role, by limiting the contact of blood elements with the subendothelial tissue of the vessel wall, and an active role in biological processes that guarantee vascular physiological integrity. The tangential force exerted by the blood flow on the endothelium (shear stress) plays a major role in the correct functioning of this tissue by regulating, through the action of mechanoreceptors, the expression of genes that control proliferation, survival, and expression of markers of the endothelial cells [24].

A functional endothelium, in conjunction with vascular smooth cells (SMCs) and pericytes, is determinant for the regulation of vascular tone by balancing the production of inducers of vasodilation, such as nitric oxide (NO), which activates cytoplasmic guanylate cyclase in vascular smooth-muscle cells and decreases vascular tone through cGMP-dependent mechanisms and prostacyclin 2 (PGI2), and vasoconstrictors, such as endothelin-1 (ET-1) and thromboxane (TxA2) [25]. Endothelin-1 (ET-1) is secreted mainly by endothelial cells, and in addition to a vasoconstrictor effect, it causes fibrosis in the vascular wall and stimulates the production of reactive oxygen species and the secretion of proinflammatory cytokines [26]. Similarly, in addition to vasoconstriction [27], TxA2 contributes to the expression of surface adhesion proteins, such as endothelial leukocyte adhesion molecule-1 (ELAM-1), intercellular adhesion molecule 1 (ICAM-1), and vascular adhesion molecule 1 (VCAM-1) [28,29], promotes endothelial cell migration and angiogenesis [30], and increases platelet aggregation contributing, together with other endothelium specific molecules, such as Krüppel-like factor (KLF) 2 and KLF 4 [31], to the regulation of the coagulation process.

**Figure 1:**
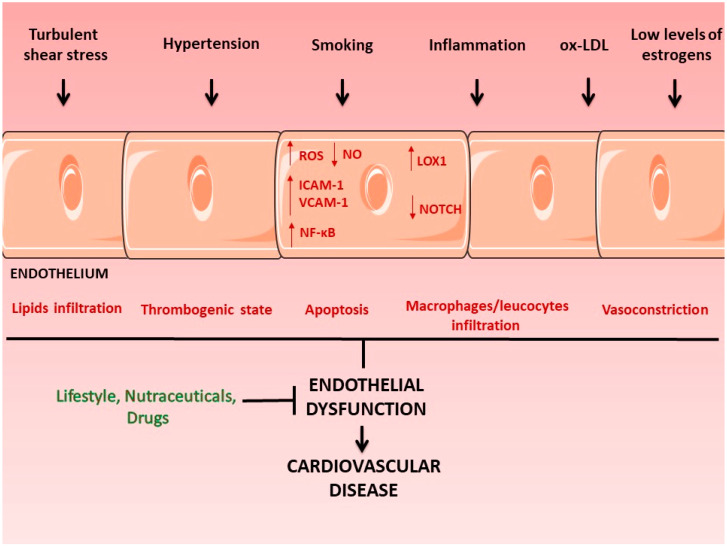
**Endothelial dysfunction and cardiovascular diseases (CVDs)**. Damage to the endothelium caused by different stimuli, including turbulent shear stress, hypertension, smoking, exposure to proinflammatory mediators, ox-LDL, and low levels of estrogens, leads to alterations in endothelial cells, such as increased production of ROS, reduced bioavailability of NO, overexpression of ICAM-1 and VCAM-1, and alteration of molecular pathways involved in endothelial dysfunction (including LOX-1, NF-κB and Notch pathway). The mechanical and biological conditions cited above cause the loss of endothelium integrity, leading to vasoconstriction, lipids, macrophages, and leukocyte infiltration in the subendothelial space, thrombogenicity, and apoptosis of endothelial cells, creating the conditions for the formation of atherosclerotic plaques, the main cause of cardiovascular disease.

Damage to the endothelium caused by different stimuli, such as exposure to proinflammatory mediators, low levels of estrogens, and oxidized low-density lipoproteins (oxLDL), leads not only to reduced bioavailability of NO but also to increased neointimal thickness (composed of proliferating SMCs, which synthesize extracellular matrix, recruited to the intimal layer), overexpression of genes coding for proteins mediating the adhesion of inflammatory cells to the endothelium, such as ICAM-1 and VCAM-1, increased production of oxygen free radicals (ROS), and increased thrombogenicity and apoptosis [32] (Figure 1). It is also important to highlight that the hemodynamic conditions to which the endothelium is exposed also determine its function. The endothelium located in bends and bifurcations, such as the aortic arch and coronaries bifurcation, is exposed to shear stress forces different from the shear stress forces present in other areas, such as the thoracic aorta (turbulent shear stress versus laminar shear stress) and this condition makes the endothelium primed for a dysfunctional state [33,34].

ox-LDL = oxidized low-density lipoproteins; ROS = reactive oxygen species; ICAM = intercellular adhesion molecule; VCAM = vascular cell adhesion molecule; NF-κB = nuclear factor kappa-light-chain-enhancer of activated B cells; LOX1 = lectin-like oxidized low-density lipoprotein receptor 1, NO = nitric oxide.

The mechanical and biological conditions cited above cause the loss of endothelium integrity, leading to lipid, macrophage, and leukocyte infiltration in the subendothelial space, creating the conditions for the formation of atherosclerotic plaques, the main cause of ischemic heart disease and peripheral artery disease. These alterations of the endothelium are also the first step toward calcific aortic valve disease, a pathological condition of the aortic valve, located between the aorta and the left ventricle, which presents an external layer of endothelial cells [35]. A large body of evidence shows that endothelial dysfunction underlies the severe manifestation of COVID-19 caused by SARS-CoV-2 [36].

Studies in heart failure patients have shown that the gravity of the disease is associated to the extent of the endothelial dysfunction [37,38,39].Therefore, endothelial dysfunction is an important prognostic factor of the gravity of the disease and its monitoring can guide the clinician in the management of the patients. The ability of the endothelium to act as a regulator of vascular tone is used in the clinic to evaluate the condition of the endothelium by injecting acetylcholine in the brachial artery of a patient: acetylcholine will induce NO production causing the relaxion (brachial artery flow-mediated vasodilation) of the artery. Other approaches today are also used to evaluate the extent of endothelial dysfunction in patients, such as a blood pressure cuff to elicit reactive hyperemia for measuring brachial wall shear stress and flow-mediated dilation [40], and in vitro by the determination of the apoptotic rate in umbilical vein endothelial cells grown in the presence of patient serum [39,41].

The functions of the endothelium can be, at least partially, restored by lifestyle modification and/or specific pharmacological approaches slowing down, or halting, the progression of the cardiovascular disease. In stable coronary artery disease patients, the use of ticagrelor, an inhibitor of P2Y 12, the receptor for adenosine diphosphate, improves endothelial function [42] and inhibits the adenosine diphosphate (ADP)-induced vasoconstriction mediated by P2Y 12 receptors in vascular smooth-muscle cells [43,44]. Similarly, ivabradine, a heart rate-reducing agent, exerts a protective effect against endothelial dysfunction by promoting favorable hemodynamic conditions (reduced turbulent shear stress) in the aortic arch [45]. Since low estrogen conditions are associated with endothelial damage, the use of agents with estrogen-like activity (such as polyphenols) could be useful in reducing endothelial dysfunction in postmenopausal women [46,47].

There is strong evidence showing the potential role of natural antioxidants in promoting endothelium health [6,48,49,50,51,52,53]. Among natural antioxidants are polyphenols, a large family of naturally occurring organic compounds characterized by several hydroxyl groups on aromatic rings, widely found in grains, cereals, vegetables, spices. and fruit. It has been shown that phenolic compounds exert a protective effect on endothelial function not only by reducing lipid disorders and hyperglycemia but also by reducing oxidative stress [54,55,56]. Resveratrol, a phytoalexin derived from many plants, is a potent scavenger of free radicals and a regulator of genes involved in endothelial dysfunction, including the gene coding for Sirtuin 1 (Sirt1), a protein deacetylase acting as an oxidative stress sensor and a repressor of inflammatory response [57], whose increase in peripheral blood mononuclear cells (PBMCs) of stable coronary disease patients with chronic obstructive pulmonary disease (COPD) treated with ticagrelor has been linked to the protective effect of this antiplatelet drug against endothelial dysfunction [42]. Among the other protective effects of resveratrol, there is the inhibition of NADPH oxidase (NOX)-mediated production of ROS by downregulation of the expression and activity of the oxidase, the prevention of superoxide production from uncoupled endothelial eNOS by upregulation of the tetrahydrobiopterin-synthesizing enzyme GTP cyclohydrolase I, and the upregulation of the nuclear factor erythroid-2-related factor 2 (Nrf2), a major regulator of the expression of antioxidant enzymes and genes related to redox homeostasis [58]. In subjects with hypercholesterolemia and low–moderate cardiovascular risk, a novel nutraceutical compound containing a low manocolin K dose, polymethoxyflavones, and antioxidants reduced endothelial dysfunction (as indicated by the reduction in endothelial cell apoptosis), improved lipid profile, and reduced oxLDL [41]. Another emerging nutraceutical compound with known antioxidant properties is berberine (BBR), a quaternary ammonium salt from the protoberberine group of isoquinoline alkaloids (5,6-dihydrodibenzoquinoliziniumderivative) found in *Berberis* spp. plants (Berberidaceae). Both preclinical studies and clinical trials have shown the protective effect of BBR against endothelial dysfunction [7]. It has been shown that in HUVEC, treatment with BBR prevents oxLDL and TNFα-induced LOX-1 expression and reduces oxidative stress, key events that lead to NOX, MAPK/Erk1/2, and NF-κB activation as indicated by the BBR-mediated reduction of NOX4-derived ROS production. This effect is due, at least partially, to the increase in adenosine monophosphate-activated protein kinase (AMPK) phosphorylation [48]. Additionally, in both cultured endothelial cells and blood vessels isolated from rat aorta, BBR induced eNOS and promoted hyperactivation of glutathione peroxidase (GSH-Px) and superoxide dismutase (SOD) in the liver of mice, attenuating H_2_O_2_-induced ROS [8]. One of the limitations of the protective effects of natural antioxidants is their low bioavailability, mostly influenced by their complex chemical structures. In the case of polyphenols, fermentation with lactic acid bacteria (LAB) seems a successful approach to overcome this limitation. This process, in fact, leads to increase bioavailability of polyphenols contained in natural products, since enzymes present in LAB produce simpler phenolic compounds that may be better absorbed in the duodenum [59]. Consistently, *Pushgay* berries fermented with LAB, but not unfermented *Pushgay* berries, reduce oxidative stress induced by menadione and protect HUVECs against H_2_O_2_-induced cell toxicity by regulating the redox state of the cell, specifically increasing the activity of glutathione reductase (GR) [48].

Many of the molecular pathways involved in endothelial dysfunction are well known, and include, together with the above cited players such as nitric oxide synthase (NO), lectin-like oxidized low-density lipoprotein receptor 1 (LOX-1) involved in the activation of nuclear factor kappa-light-chain-enhancer of activated B cells (NF-κB) pathway [60,61], emerging pathways such as those related to loss of function of the protein KRIT1 [62], and the epidermal growth factor receptor (EGFR) [43]. Recently, pathways known to regulate the development of the cardiovascular system, such as Notch [63,64,65,66], Hedgehog [67], and Wnt [68], have been identified as major determinant of the health of the endothelium and new pathways are being discovered [69].This knowledge will be crucial to identify novel therapeutic targets for patients that do not respond to commonly used treatments or become resistant to them and to identify bioactive molecules able to prevent/counteract endothelial dysfunction and related pathologies.

## 4. Apple

According to the Food and Agriculture Organization (FAO), apple production has increased by about 34% in the last 15 years (2006–2020). It was estimated to be about 64 × 10^6^ tons in 2006, while estimation for 2020 was about 86 × 10^6^ tons [70]. China, the USA and Turkey are the largest-producing countries, followed by Iran, India and Italy. In 2020, Italy produced about 2.5 × 10^6^ tons, accounting for about 15.5% of the entire European production [70]. Even though most apple production is destinated to be consumed as it is, about 11.6 × 10^6^ tons/year worldwide is processed to obtain apple products, leading to 3.5 × 10^6^ tons of waste and by-products.

### 4.1. Exploiting Apple Pomace

Apple pomace is the by-product generated from juice pressing in the production of apple juice and cider. It is a solid mass consisting of a mixture of peel, seed, stem, and pulp residues, and represents up to 30% of the fruit weight. Apple pomace is mainly a source of dietary fiber (50% of dry pomace weight) and phenolic compounds (up to 4 g/kg dry weight) [71]. Apple pomace is still used for animal feeding, and it has very low protein content, which is needed to support animal growth, but several other industrial and agronomical applications have been proposed to exploit these by-products in a circular economy perspective [72]. In the last 25 years, apple pomace has been used to produce several products, such as enzymes, polygalacturonases or hydrolytic depolymerases, involved in the degradation of pectic substances. It has also been applied in food and textile processing [73,74]. Apple pomace has been used as a substrate for bioethanol production [75] and biopolymer synthesis, such as fungal chitosan [72]. More recently, further applications have been proposed for apple pomace. Bioactive compounds from apple waste have been obtained with different extraction methods to be used as food ingredients, in the production of cosmetics, and for their beneficial effects as anti-inflammatory and antioxidant agents in the management of insulin resistance and blood lipid profile [76,77,78,79,80].

Since 2010, chemists have proposed new extraction approaches to improve polyphenol yield from apple pomace, quicken extraction time, and limit the need for organic solvents and purification steps [81]. Attention toward more sustainable and environmentally friendly extraction procedures has pushed the research of new extraction techniques [81,82,83]. Virot et al. in 2010 [82], proposed ultrasound-assisted extraction as a potential method to obtain apple pomace polyphenols. Comparing their procedure to conventional extraction without sonication, they describe their method as able to improve polyphenol yields, more sustainable, less time- and energy-consuming and suitable for upscaling at the industrial level. In terms of yield, ultrasound-assisted extraction led to a +20% catechin extraction, flavan-3-ols and procyanidins increased by 25%, and dihydrochalcones and flavonols by about 5%. In apple pomace, due to both covalent and noncovalent interactions and links with saccharides, polyphenols may result less extractable by traditional methods with aqueous/organic solvents [84]. For this reason, and to improve extraction yields, some authors have applied microwave superheated water technology to apple pomace [85]. With this technique, the polyphenol yield was 5 g/kg dry pomace, with flavan-3-ols, flavonols and dihydrochalcones as the three most present phenol classes [83]. Pulsed electric fields have also been proposed to improve polyphenol yield by increasing cell-membrane permeability through the formation of pores. Advantages of this technique are the reduced extraction time, the possibility to realize bound phenols, leading to a higher total phenol yield, and the low thermal effect [86]. A further extraction approach is represented by enzymatic-assisted extraction. In apple pomace polyphenol-extraction studies, the use of enzymes like endoxylanase and endo-β-1,4-glucanase have been demonstrated to increase phenol yield; however a long time and temperature up to 40 °C are needed [87]. In 2018, Zhang et al. [88] proposed a combination of enzymatic-assisted extraction and ultrasound, defining the optimal extraction conditions to obtain a higher phenol yield. A complete and exhaustive description of polyphenol-extraction methods from apple pomace has been published by Wang et al. [81].

### 4.2. Cardiovascular Protective Effects of Bioactive Compounds from Apple By-Products

Cardiovascular diseases (CVDs), a wide group of pathologies including atherosclerosis, stroke, cerebrovascular disease, and coronary heart disease, still represent the first cause of death worldwide [89]. Dietary patterns rich in fruit and vegetables providing a significant amount of fiber and phenolic compounds correlate with a lower risk for developing CVDs and CVD mortality. [90]. Clinical trials on apple intake show that apple consumption decrease LDL cholesterol levels [91], improve endothelial function [92], and decrease the body mass index [93].

Phenolic bioactive molecules mediate cardiovascular protection by different mechanisms, such as direct and indirect antioxidant effect [5], anti-inflammatory activity [94], and maintenance of nitric oxide (NO) homeostasis by modulating endothelial nitric oxide synthase (eNOS) activity.

Waldbauer et al. [95] evaluated the ability of polar and apolar fractions of apple pomace methanol/water extracts to modulate eNOS activity in a human endothelium-derived cell line (EA.hy926). They found that the mixture of triterpenoic acids, instead of total phenolic fraction content, was directly related to eNOS activation, suggesting that apple pomace extracts can improve endothelial function. In further research, both on human vein endothelial cell (HUVEC) cultures and in a human clinical trial, the effect of apple polyphenols on vascular oxidative stress and endothelial function was studied [5]. Cell culture studies demonstrated the ability of glycosylated phloridzin, chlorogenic acid and quercetin from apple skin to counteract uric acid production in a dose-dependent manner by inhibiting xanthine oxidase. Moreover, the clinical trial demonstrated that 300 mg/day of apple polyphenols for 8 weeks reduced fasting glycemia, serum uric acid and improved endothelial reactivity [5]. Other studies have demonstrated the possibility to modulate glucose absorption, to reduce postprandial glycaemia and insulinemia by phloridzin, chlorogenic acid and quercetin treatment. Phloridzin and quercetin are known to inhibit, in vivo, the intestinal glucose transporters SGLT1 and SGLT2 [5,96,97].

Apple phenols have been claimed to exert cardiovascular protective effects by acting as antioxidants. In fact, phloridzin and quercetin are known to exert indirect antioxidant effects by inducing gene expression of phase-2 detoxifying and antioxidant enzymes [98,99]. Quercetin is known to protect cardiac and endothelial cells from oxidative stress, and in cardiac cells quercetin was demonstrated to upregulate gene expression of phase 2 enzymes, highlighting its ability to act with an indirect antioxidant mechanism, and to modulate PI3K/Akt and ERK1/2 signaling pathways [100,101]. In a model system represented by human brain, microvascular endothelial cells exposed to hypoxia and reoxygenation, quercetin exerted its antioxidant effect by activating the nuclear erythroid 2-related factor 2 (Nrf2) signaling pathway [102].

Nrf2 induction was also the molecular target involved in apple pomace extracts that induced antioxidant effect in a mouse model of liver oxidative stress [103].

Inflammation and oxidative stress have been defined as “inseparable partners” not only because the chronic or excessive activation of one of the two phenomena inevitably determines the activation of the other but also because the molecular mechanisms and pathways involved cannot be untangled and they share a mutual signaling cascade [104]. Apple polyphenols exhibit anti-inflammatory activity not only in in vitro studies but also in animal models and in clinical trials of chronic inflammatory diseases [105]. In obese subjects, postprandial apple intake reduced plasma level of proinflammatory cytokines IFN-γ, IL-6, and TNF-α [106]. In vitro, quercetin exerted an anti-inflammatory effect by inhibiting LPS-induced iNOS protein expression and subsequent NO overproduction in cardiomyoblasts (H9C2 cell line) [107]. However, administration of quercetin (500 mg/day) to post-myocardial infarction patients did not result in a significant reduction of proinflammatory biomarkers [108], highlighting that administration of a single purified bioactive compound does not always provide positive effects. Eleven pentacyclic triterpenes have been detected in apple pomace [95], and among them, tormentic and ursolic acid have been shown to reduce inflammation by inhibiting NF-κB signaling [109,110].

Apple pomace is not merely a source of phenols—50% of its mass is represented by dietary fiber. Even though fibers cannot be considered bioactive compounds, they play a role in the prevention of CVD-related risk factors [111]. Apple fibers are both insoluble, e.g., cellulose and hemicellulose, and soluble, e.g., pectin, which in turn contains homogalacturonans and rhamnogalacturonans. Galacturonic acid residues are partially esterified, methylated, and the degree of methylation appears to modulate viscosity and increase the amount of cholesterol eliminated through the feces [112,113]. Before being fermented into short-chain fatty acids by gut microbiota, apple pectin modulates bile acid enterohepatic circulation and reduces plasma cholesterol [114,115].

An apple cholesterol-lowering effect has also been investigated in human studies. Ravn-Haren et al. (2013) evaluated the effect of daily apple, apple pomace, or juice consumption for 4 weeks on total and LDL cholesterol levels in a small group of healthy subjects. Even if nonsignificant, after the dietary treatment period they observed a trend of cholesterol reduction [116]. More recently, Koutsos et al. [117] evaluated in 40 human subjects the cholesterol-lowering effect of the consumption of two apples/day for 8 weeks and demonstrated that the treatment was effective in reducing serum, total and LDL cholesterol, and triglycerides.

**Figure 2:**
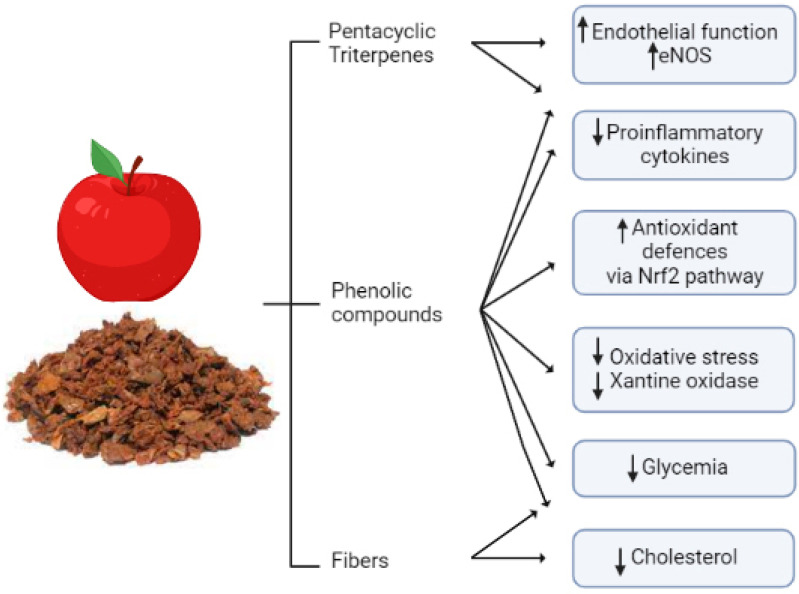
**Apple pomace classes of bioactive compounds and their cardio- and vasculoprotective actions.** Apple pomace contains fibers that mainly provide cholesterol-lowering effects, but it also contains bioactive compounds such as phenols and triterpenes that provide multiple effects positively affecting the cardiovascular system, such as improvement in endothelial function and antioxidant effect, reduction in proinflammatory cytokines and oxidative stress, and a hypoglycemic effect. Overall, thanks to its composition, apple pomace exerts pleiotropic cardio- and vasculoprotective effects by targeting multiple signaling pathways and risk factors. The figure is original and created with BioRender.com.

Apple fibers are not the only apple component responsible for reducing cholesterol levels. They may both bind directly to bile acids, increasing their excretion in feces and activating hepatic cholesterol 7alpha-hydroxylase increasing cholesterol excretion, as was suggested by supplementation studies in an animal model [118]. A representative summary of the cardioprotective role of apple pomace is reported in Figure 2.

## 5. Pear

Data from the FAO describe that worldwide pear production increased by 15.6% between 2006 and 2020. Most recent available data show that world pear production was about 23 × 10^6^ tons in 2020, with China, Italy, and the USA the largest-producing countries. In the last 15 years, pear production in Italy has decreased by about 32%; however, Italy is still the top producer in Europe and the second worldwide [70]. It is one of the most important stone fruits and widely consumed as fresh when fully mature and/or in processed forms, such as in purees and jam. The other pear parts, such as peels, seeds, and leaves, are usually discarded.

### 5.1. Exploiting Pear Pomaces

As well as apple pomace, pear pomace is the by-product generated from pear processing and it still contains bioactive compounds, carbohydrates and fibers that can be exploited at the industrial level. Possible applications of pear pomace have been proposed: as a substrate for bacterial cellulose and pear vinegar coproduction [119], as a source of fiber and antioxidants for the prevention of obesity and oxidative stress in animal studies [120,121].

The analyses of phenolic compounds in different anatomical pear parts demonstrated that pulps contain the lowest concentration of phenolic bioactive molecules, which, in turn, are enriched in seeds and peels [122]. For this reason, pear pomace appears a good source of these bioactive compounds. Characterization of pear bioactive molecules has been carried out, demonstrating the presence of polyphenols such as phenolic acids and flavonoids, and triterpenes [123]. In 2014, Li et al. [124] analyzed the chemical composition of peels and pulp from 10 pear varieties. They found that, both in peel and pulp, the prevailing monomeric compounds were arbutin, oleanolic acid, ursolic acid, chlorogenic acid, epicatechin, and rutin. Moreover, quantitative analyses of total phenols, flavonoids, and triterpenes content revealed that all compounds detected were 6–20 times more concentrated in the peel than in pulp [124], suggesting that in the pear-processing food chain most bioactive compounds are discarded in pear pomace.

### 5.2. Cardiovascular Protective Effects of Bioactive Compounds from Pear By-Products

Pear bioactive molecules have been claimed to exert multiple healthy effects. At the cardiovascular level, they might be beneficial by positively modulating numerous risk factors for cardiovascular diseases. Chlorogenic acids have been demonstrated to exert anti-inflammatory properties by downregulating TNF-α and IL-8 in cell culture studies [125]. Pyrus pashia, known as the Himalayan pear, extracts, rich in chlorogenic acid, catechin, epicatechin, and arbutin, inhibited 5-lipoxygenase, cyclooxygenase-2 activities and reduced IL-6 and TNF-α expression in vitro. Moreover, these extracts demonstrated similar anti-inflammatory properties in an in vivo mouse model [126]. Oleanolic acid, a pentacyclic triterpenoid detected in pear peels, is known to exhibit anti-inflammatory properties. In a model system of carotid artery-injured diabetic rats, oleanolic acid suppressed the inflammasome signaling pathways and reduced serum levels of TNF-α, IL-1β, IL-6 and IL-18 [127]. A recent study on olive oil pomace, which is also rich in oleanolic acid, highlighted the anti-inflammatory properties of this triterpenoid [128].

Studies focused on arbutin biological effects showed its antioxidant and anti-inflammatory properties. In cardiac cells (H9C2), arbutin pretreatment protected cells from isoproterenol-induced oxidative stress [129]. Moreover, in an in vivo model system of cardiac damage induced by lipopolysaccharide in rats, arbutin treatment reduced inflammation by modulating TNF-α and IL-6 levels [130].

Pear peel components, such as chlorogenic acid, have been proposed to exert direct effects on the vascular endothelium. Ex vivo and in vitro studies showed that chlorogenic acid improved NO production at the vascular level and partially inhibited angiotensin converting enzyme (ACE)-I [131,132].

Pear and pear by-product extracts have also shown antihyperglycemic properties. Both in rats and diabetic mice, pear peel extracts reduced fasting glycemia. Different mechanisms have been proposed to explain this effect: on one side, these extracts seem to slow down carbohydrate digestion by inhibiting alpha-amylase and alpha-glucosidase activity; on the other, they have been claimed to stimulate pancreas insulin secretion [133,134].

**Figure 3:**
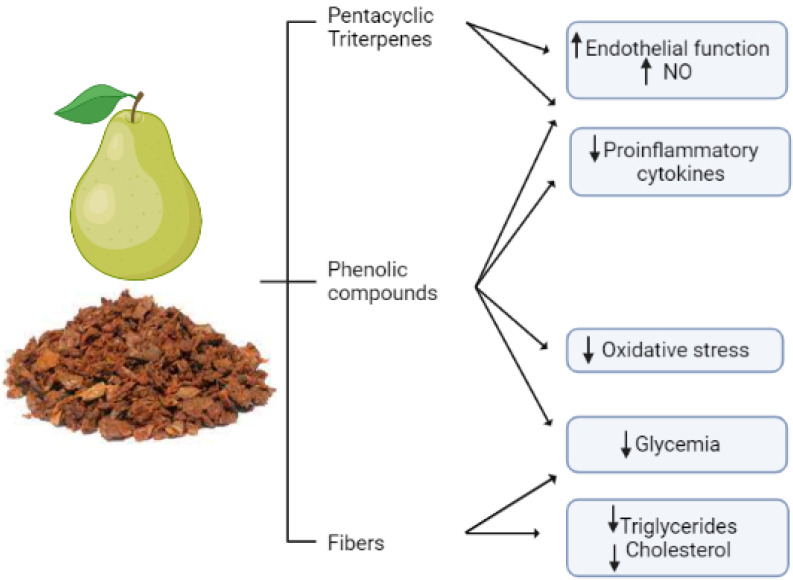
**Pear pomace classes of bioactive compounds and their cardio- and vasculoprotective actions.** Pear pomace contains fibers that mainly provide cholesterol- and triglyceride-lowering effects, but it also contains bioactive compounds, such as phenols and triterpenes, that provide multiple effects positively affecting the cardiovascular system, such as improvement in endothelial function, reduction in proinflammatory cytokines and oxidative stress, and a hypoglycemic effect. Overall, thanks to its composition, pear pomace exerts pleiotropic cardio- and vasculoprotective effect by targeting multiple signaling pathways and risk factors. The figure is original and created with BioRender.com.

Pear peal extracts have also been claimed to modulate hyperlipidemia, a further risk factor for CVDs. In a mouse model, this extract lowered triglycerides, total cholesterol, and LDL [134]. A representative summary of the cardioprotective role of pear pomace is reported in Figure 3.

## 6. Sugar Beet

Sugar beet (Beta Vulgaris variety Saccharifera) is one of the most important crops, along with cane, for sugar production in Europe [135]. Nowadays, 120 million tons of beets are produced in Europe, from which about 16 million tons of sugar are extracted (50% of European production of sugar). Even if sugar beet is one of the traditional crops produced in Italy, only 30% of its national demand is covered, while 70% comes from abroad, mainly from France and Germany, which are the leading producers. The major sugar beet-producing regions in Italy are Emilia-Romagna, Veneto, Puglia, Lombardy, and Marche.

The cultivation of beet is one of the most virtuous in terms of greenhouse gas emissions and it is strategically relevant in agricultural rotation thanks to its ability to release organic nutrients and minerals into the soil. It is considered an “improving” crop capable of enriching fields after harvest, increasing their production yield, and “renewal” for tillage and fertilization at the beginning of the rotational cycle. When introduced into the rotation process, it significantly reduces the environmental impact of farming [136,137]. As such, the sugar beet plant has not just the peculiar characteristic of accumulating sucrose in the root system but can be seen as a fundamental crop for agricultural sustainability. Therefore, this plant is of particular interest not just from an economic point of view.

### 6.1. Sugar Beet Waste and By-Products

**Figure 4:**
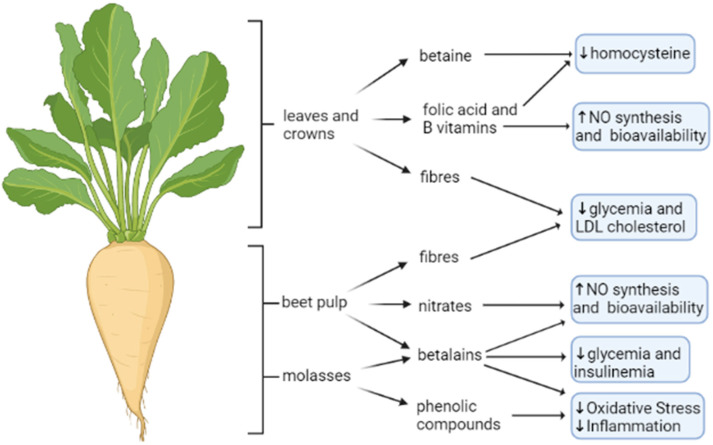
**Sugar beet bioactive compounds and their cardio- and vasculoprotective actions.** The entire sugar beet plant is a source of bioactive molecules with a potential cardio- and vasculoprotective role. Betaine and B vitamins from leaves and crown are involved respectively in homocysteine detoxification and NO synthesis. Nitrates, betalains and phenolic compounds from beet pulp, and its by-product molasses, are involved in NO bioavailability, glycemia control and reduction in oxidative stress and inflammation. Moreover, fibers from different plant parts exert hypoglycemic and cholesterol-lowering effects. The figure is original and created with BioRender.com.

The primary aim of growing sugar beet is the production of refined sugar. However, any other by-products that remain or are produced in the manufacture of refined sugar from beets, i.e., beet tops (leaves and crowns), pulp, and waste molasses, might have a much greater commercial value than the original one. Right now, the alcohol made from waste molasses and commercial fertilizers made from refuse slop represents an effective “upcycling” way to convert a by-product into a high-added-value one in economic terms. Attempts are also being made to use beet leaves in the production of methanol, so general interest may be taken in the proper utilization of the sugar beet by-products. Here, we discuss the main bioactive molecules present in sugar beet products and their possible beneficial role in endothelial function (Figure 4).

The first by-product obtained in sugar beet processing is the top, made of leaves and crowns, which is removed by the grower in preparing the beets for the factory at harvest time. Although the sugar is produced in the leaves, it constantly passes into the root, where it is stored. Sugar beet leaves also contain betaine, fiber, folic acid, and B vitamins [138].

Beet leaves have a huge quantity of the amino acid betaine, used by plants as an organic osmolyte to protect against osmotic stress, aridity, high salinity, or temperature changes [139]. The intracellular accumulation of betaine, by not disturbing enzyme functions, protein structure, or membrane integrity, allows water retention in cells, protecting them from the effects of dehydration. Betaine is also a methyl-group donor, used by cells as a cofactor in the biochemical process called “transmethylation” which is essential for cellular metabolism. Betaine plays a key physiological role in the process of detoxifying homocysteine (Hcy), a powerful oxidant, into methionine. Recently, a QuEChERS (quick, easy, cheap, rugged and safe) procedure was successfully applied for the isolation and quantification of betaine in different beetroot parts (peel, juice, pulp), revealing betaine levels from 2 to 5 mg/g according to the plant fraction [140].

The sheer volume of extracted waste from sugar production is molasses and pulp. Indeed, just the fresh pulp constitutes about 80% of the weight of the beets, and following the extraction of sucrose, the pulp is separated off and frequently used worldwide in animal feed or converted into biogas, while molasses are recycled for livestock feed and alcohol-based preparations [141,142]. Fibers in sugar beet root crops are not extensively lignified; generally, they comprise approximately one-third pectin, one-third hemicellulose, and one-third cellulose, so these by-products can have functional characteristics due to their significant fiber content, both soluble and insoluble, which can provide interesting technological properties for the food industry, such as enrichment of pasta, cakes, and cookies [143,144].

High amounts of antioxidant molecules are found in sugar beet molasses [145], which means that this by-product could be used as a raw material for the production of functional foods, nutraceuticals, and supplements. Molasses is the by-product that remains after the crystallizable sugar has been separated from the concentrated beet juice, so it contains nearly 50% of sugar that cannot be separated from the non-sugars by ordinary methods, owing to the presence of various salts that have been taken up by the beet from the soil in the process of growth [146]. Chen and coworkers [145], recently observed through HPLC-DAD-MS/MS a high quantity of anthocyanins (cyanidin-3-O-rutinoside, cyanidin-3-O-glucoside, delphinidin-3-O-rutinoside, and delphinidin-3-O-glucuronide) in sugar beet molasses extracted by an eco-sustainable and chemical-free ultrasound-assisted process (UAE). They also detected other functional components with known antioxidant properties, such as gallic acid, vanillin, hydroxybenzoic acid, syringic acid, catechin, and ferulic acid.

Betalains are water-soluble, nitrogen-containing, red, and yellow betalamic acid-derived pigments with two classes of compounds: betacyanin (red-violet) and betaxanthin (yellow-orange). Betalains are relatively sensitive to environmental factors, such as heat and high or low pH (stability pH range of 3 to 7) and oxygen, which can cause degradation of the metabolites during the processing of plant material [147], but more stable than anthocyanins, which have an optimum pH range of 5 to 6 [148].

Even if red beetroot has the highest levels of betalains, sugar beet has an appreciable amount of them [149]. Betacyanins are derivatives of betanidin (or betanin), an iminium adduct of betalamic acid and cyclo-DOPA, whereas betaxanthins result from the condensation of α-amino acids or amines with betalamic acid [150]. Low-temperature enzyme-assisted extraction has been recently applied to isolate betalains from red beets, opening new possibilities to also apply the method to sugar beet by-products [151]. Betacyanins and betaxanthins, both present in sugar beet [149], have been shown to have cardiovascular disease protection [152,153].

### 6.2. Cardiovascular Protective Effects of Bioactive Compounds from Sugar Beet By-Products

Sugar beet by-products are an interesting source of bioactive compounds that could be potentially applied in the prevention of cardiovascular diseases.

One of the most studied is betaine, due to its role as an HCy detoxifier. It is known that large amounts of HCy in the body are toxic, so almost 50% of the Hcy is recycled in the liver, using betaine as a methyl-group donor. The other half is detoxified through an additional process (transmethylation): 5-methyl-tetra-hydrofolate, produced by folic acid also present in beet tops, gives up its methyl group to Hcy by converting it to methionine through methionine synthase (vitamin B12). Methionine, in turn, is converted to S-adenosyl methionine (SAM), through methionine adenosyltransferase, a coenzyme involved in the transfer of methyl groups to various biological substrates, such as nucleic acids, proteins, and lipids. Many epidemiological studies have confirmed that elevated plasma levels of homocysteine (Hcy) are associated with an increased risk of vascular disease, including endothelial dysfunction, proliferation of smooth-muscle cells, increased oxidative stress, reduced activity of glutathione peroxidase, and promoting inflammation [154]. Epidemiological studies showed that Hcy is associated with premature peripheral, coronary artery and cerebrovascular disease independently of other risk factors. Clinically, this manifests as impaired flow-mediated vasodilation and is mainly due to a reduction in nitric oxide (NO) synthesis and bioavailability. The effect of impaired NO release can in turn trigger and potentiate atherothrombogenesis and oxidative stress [155].

In this regard, betaine and folic acid supplementation could reduce Hcy levels, thus reducing vascular disease, even if the mechanism is not fully elucidated [156]. Recent studies suggest an indirect mechanism for Hcy toxicity that is secondary to its precursor S adenosylhomocysteine (SAH) accumulation [157,158], while others reported that the association between Hcy and cardiovascular disease may be explained by low SAM levels or a low SAM/SAH ratio [159,160]. Indeed, low plasma levels of SAM have been associated with endothelial injury and atherosclerosis [161], and SAM treatment prevents endothelial dysfunction in HFD-fed animals by inducing HO-1 in vascular endothelial cells, preventing cell apoptosis [162]. SAM has been found to be useful in both the prevention and treatment of several metabolic disorders, including diabetes [163,164].

As far as sugar beet fiber derived from sugar production waste pulp is concerned, its inclusion in the diet of healthy volunteers resulted in significant physiological changes, such as reductions in the levels of both postprandial plasma glucose and blood LDL cholesterol, effects confirmed by the European Food Safety Agency (EFSA) [165]. Leontowicz M. et al. demonstrated that sugar beet pulp fiber and to a lesser degree apple pomace fiber possess hypolipidemic properties in rats fed a high-cholesterol diet [166]. As such, sugar beet fibers may be used as a palatable, fibrous food ingredient for human consumption [167].

Pectins from sugar beet pulp have been seen to be important modulators of gut microbiota composition: in vitro studies on a system simulating the microbiota of normal-weight and obese subjects showed that sugar beet pectins promote the growth of *Clostridium* spp. cluster XIV, which can lead to reduced circulation of inflammatory markers as well as reduced body weight [168]. The prebiotic effect of sugar beet pectins has also been demonstrated by stimulating the growth of *Faecalibacterium prausnitzii*, which is considered an anti-inflammatory species that can stimulate butyrate production [169]. Of note, in the microbiota of obese subjects, this bacterium is scarcely present and sugar beet pectins promote its growth and colonization [168].

Additionally, beet consumption can be considered an adjuvant therapeutic option in a range of pathologies associated with oxidative stress and inflammation. These possible properties and applications have been primarily associated with the presence of antioxidant molecules in sugar beet, i.e., betalains and polyphenols, which have recognized anti-inflammatory activities shown in in vitro and in vivo experiments [170]. Indeed, beetroot peel powder (BPP) was used to obtain also the value-added antioxidant mayonnaise with high betalain (1.18 ± 0.03 mg/g DW) and polyphenolic (225.36 ± 1.97 mg GAE/g DW) content [171].

Among betacyanins, betanin (betanidin 5-O-β-D-glucoside) is the most representative phytochemical. It is a water-soluble nitrogenated heterocyclic compound capable of inhibiting lipid membrane and low-density lipoprotein (LDL) peroxidation, modulating ROS generation and gene expression in order to reduce inflammatory cytokine release and increasing antioxidant enzyme activities [172,173]. In vascular tissue, the antiradical activity of betanin maintains endothelial function and reduces the atherogenesis process. In addition, betanin can modulate redox-mediated signal-transduction pathways involved in inflammation responses in endothelial cells by inhibiting the intercellular cell adhesion molecule-1 (ICAM-1) [174].

Betanin also regulates liver glucose metabolism-related enzymes in diabetes type II, such as those involved in the glycolytic pathways, like glucokinase, glucose-6-phosphatase, pyruvate kinase, in the pentose phosphate pathway, i.e., glucose-6-phosphate dehydrogenase, and in gluconeogenesis, like fructose-1,6-bisphosphatase [174]. Chronic hyperglycemia promotes tissue fibrosis mediated by advanced glycation end products (AGEs) and transforming growth factor-beta (TGF-β). The antidiabetic role of betanin has been proven to revert hyperglycemia, hyperinsulinemia, insulin resistance, and glycation products in rats induced to experimental diabetes by high fructose intake, streptozotocin–nicotinamide, or a high-fat hypercaloric diet [174].

Therefore, the effects of betanin on inflammation, oxidative stress, endothelial function and diabetes are well documented [173], even if more studies are necessary to confirm the safety and efficacy of sugar beet pulp and molasses as supplements in human pilot studies.

Even if betacyanins have a well-documented effect on CVD prevention, betaxanthins show greater bioavailability [175]. Owing to its high bioavailability and health-protective effect, betaxanthin can be employed as a food supplement not just for its beneficial effects but also to enhance the quality of processed food products [175].

Indeed, even if few studies are present in the literature with respect to betanin, indicaxanthin, a bioavailable betaxanthin with anti-inflammatory activity, can exert remarkable protective effects in an in vitro model of vascular inflammation at nutritionally relevant concentrations. Considering its ability to modulate specific endothelial genes involved in leukocyte adhesion and cholesterol transport through a redox-dependent, NF-κB-mediated mechanism, indicaxanthin might be further investigated in vivo as a potential endothelial protective agent of dietary origin [176].

Beetroot can go as deep as 3 m, allowing the plant to absorb nutrient-essential nitrogen compounds, so are enriched not only in betalains but also in nitrate (NO3-). Epidemiological studies have demonstrated that dietary (NO3−) from certain vegetables can provide a physiological substrate to produce nitric oxide (NO) that, in turn, supports cardiovascular function, causes vasodilation, and decreases blood pressure [174]. A dietary intake superior to 6.3 mmol is necessary to increase NO levels and blood pressure reductions in both healthy individuals and those presenting cardiovascular disease (CVD) risk factors.

Even if red beetroot is considered the most common NO3− source, sugar beetroot has an appreciable amount of this molecule [177]. Dietary NO3− is well absorbed in the upper gastrointestinal tract. About 25% of dietary NO3− is captured by the salivary glands, where it is reduced to NO2− by commensal bacteria that express and secret NO3−-reductase enzyme in saliva [174]. The metabolic activity of the hundreds of commensal bacteria species (i.e., Granulicatella, Actinomyces, Veillonella, Prevotella, Neisseria, Haemophilus, and Rothia genera) localized on the tongue can directly regulate NO3− to NO metabolism. Individuals with a higher abundance of NO3−-reducing bacteria can generate more salivary NO2− and, consequently, NO at a faster rate following the ingestion of dietary NO3− [178]. As such, oral NO3−-reducing microbiota is beneficial to the host and participates in the control of NO-related vasodilation. In this regard, antibiotic use or mouth rinsing with an antibacterial mouthwash can decrease NO3− conversion in NO2− [179]. After the conversion of dietary NO3− to NO2− in the oral cavity, the NO2− in the saliva is swallowed and reaches the stomach, where NO2− is nonenzymatically decomposed into NO and other bioactive nitrogen oxides in this acidic environment by vitamin C or polyphenols [174].

NO is a low-molecular-weight compound (30.01 g/mol) with a short life (from 5 to 10 s) produced in gas form, containing 11 electrons in its valence shell with an unpaired electron. This radical character confers high reactivity to this compound, since it rapidly oxidizes to NO2− and NO3−. NO displays an affinity for lipophilic environments and accumulates in the lipid milieu, such as cell membranes and lipoproteins. In human physiology, NO can exert antioxidant functions and is considered a secondary messenger, acting on the vascular endothelium, central and peripheral neurons, and immune system, inhibiting platelet activation, adhesion, and aggregation, modulating vascular tone, and improving human skeletal muscle function [174]. Interestingly, multiple pathways are used by NO to promote these actions, which depend on the cell tissue and the amount of produced NO. Indeed, both endothelium- and platelet-derived NO prevent platelet aggregation and fibrin formation, inhibiting the spread of thrombi generation, in different ways [180]. NO regulates vascular tone by diffusing across endothelial cells, reaching vascular smooth-muscle cells and, through sGC, activating the sarcoplasmic Ca2+ pump, decreasing intracellular Ca2+ and promoting vasodilation because of diminished vascular tone [181]. Moreover, it promotes the phosphorylation of the thromboxane-2 receptor and downregulates P-selectin expression, preventing platelet activation and adhesion. In addition, NO modulates fibrinogen binding via the glycoprotein IIb and IIIa (GPIIb/IIIa) receptor, increasing the dissociation constant of this receptor by fibrinogen, reducing the total number of GPIIb/IIIa receptors on the platelet surface, resulting in unfavorable conditions for platelet aggregation. Furthermore, NO stimulates tyrosine nitrosylation in the ONOO− pathway, thereby inhibiting thromboxane-2 synthesis [182]. As mentioned previously, NO’s free-radical scavenging reduces ROS, promoting cardioprotective effects on the atherosclerotic process by preventing LDL cholesterol oxidation, and reducing reactive nitrogen species (RNS) production rates [183].

As a cost-effective strategy, chronic and acute beetroot juice supplementation was proposed to help in controlling diabetes and insulin hemostasis, blood pressure and vascular function, renal health, and a possible effect on microbiome abundance. Moreover, persistent consumption of beetroot juice effectively postpones the postprandial glycemic response and decreases the blood glucose peak. The significant blood pressure-lowering effect has been seen among normotensive subjects, and tends to be more considerable among hypertensive individuals and progressive among overweight adults [184].

However, red beetroot juice used for supplementation has an NO3− concentration lower when compared to other beetroot formulations [174]. Indeed, it must be consumed in large amounts to reach an effective concentration. As such, the development of smart beetroot formulations is required, allowing production of inexpensive and attractive dietary NO3− supplements to stimulate NO production and promote beneficial health effects [174]. A representative summary of the cardioprotective role of sugar beet by-products is reported in Figure 4**.**

## 7. Conclusions

In this review, we summarized the main applications of waste and by-products from apple, pear, and sugar beet in the maintenance of endothelial function and in the prevention of cardiovascular disease. The wastes are biomass with high added value. When properly processed, they are a real reservoir of bioactive molecules with a health-promoting action, even at the cardiovascular level. These effects can be attributed to the presence of different molecules: fibers that act with a cholesterol-lowering and hypoglycemic action, phenolic compounds and triterpenes that exert antioxidant and anti-inflammatory activities, but also betaine, betalains, and nitrates, from sugar beet, which exert, respectively, detoxifying and hypoglycemic effects and behave as NO donors.

The limits of the applications of these agents are related to their complex metabolism and low tissue distribution. Indeed, it is very well known that small compounds such as phenolic acids and nitrates are more easily absorbed through the gut barrier than high-molecular-weight polyphenols [185]. However, larger molecules such as anthocyanins and betalains can be metabolized in shorter molecules through the gut microbiota [186,187], thus increasing their bioaccessibility. Even if some human pilot studies are necessary to better understand the metabolism and tissue distribution of bioactive compounds from apple, pear, and sugar beet waste matrices, data shown in the literature are promising. Overall, apple, pear, and sugar beet by-products represent a high-value bio-product source that could be valorized instead of being just used as biomass in energy production, or for animal feeding, so reducing greenhouse gas production and consequently negative impacts on human health. Instead, the possibility to exploit these wastes in the health industry can lead to economic and environmental benefits, so contributing to UN Sustainable Development Goals of the 2030 Agenda.
